# Ultrasound guided repositioning of a new suture-method catheter for adductor canal block – a randomized pilot study in healthy volunteers

**DOI:** 10.1186/s12871-018-0615-4

**Published:** 2018-10-24

**Authors:** Zarah Maria Jordahn, Tobias Stenbjerg Lyngeraa, Ulrik Grevstad, Christian Rothe, Lars Hyldborg Lundstrøm, Kai Henrik Wiborg Lange

**Affiliations:** 1 Department of Anaesthesia and Intensive Care Medicine, Copenhagen University Hospital, Nordsjællands Hospital, Dyrehavevej 29, 3400 Hillerød, Denmark; 2 Department of Anaesthesia and Intensive Care Medicine, Copenhagen University Hospital, Gentofte Hospital, Kildegårdsvej 28, 2900 Hellerup, Denmark

**Keywords:** Adductor canal block, Continuous peripheral nerve block, Perineural catheter, Catheter insertion technique

## Abstract

**Background:**

We performed a randomized, blinded pilot study in 12 volunteers to assess the feasibility to reposition an intentionally displaced suture-method catheter for two different insertion techniques for adductor canal block.

**Methods:**

Each volunteer had an ultrasound-guided suture-method catheter placed in the adductor canal (AC) in both legs. The catheters were placed using a perpendicular technique in one leg and a parallel technique in the other leg, according to randomization. 15 mL lidocaine 1% (LA) was injected in each catheter. Successful primary placement was defined as combined LA spread within the AC and loss of cold sensation 15 min after injection. All catheters were intentionally displaced, and subsequently repositioned using ultrasound. Another dose of lidocaine (15 mL 1%) was injected through the catheters and assessed for successful repositioning.

**Results:**

Successful primary placement was achieved in 83% (95% CI 55–95%) of catheters placed perpendicular to the AC, and in 75% (95% CI 47–91%) of catheters placed parallel to the AC.

Of those with successful primary placement, 100% (95% CI 72–100%) of catheters placed perpendicular to the AC, and 67% (95% CI 35–88%)) placed parallel to the AC could be repositioned.

**Conclusions:**

Placement and secondary repositioning after displacement of a suture-method catheter within the adductor canal is achievable. A perpendicular technique seems more reliable.

**Trial registration:**

NCT03315481 clinicaltrials.gov. The study was submitted on March 1, 2017. Due to clerical error, the study was posted on October 20, 2017.

**Electronic supplementary material:**

The online version of this article (10.1186/s12871-018-0615-4) contains supplementary material, which is available to authorized users.

## Background

Pain is a major concern for patients undergoing surgery [1, 2]. More than 25% of patients undergoing total knee arthroplasty (TKA) experience severe postoperative pain, numerical rating scale (NRS) above 8, on the first day after surgery [3] and approximately 50% report moderate-to-severe pain on the third day after surgery [1, 4].

Using peripheral nerve blocks as part of a multimodal approach to alleviate postoperative pain has been proposed [5] and continuous peripheral nerve blocks (CPNB) seem superior to single-injection nerve blocks, particularly in the context of orthopedic surgery [6].

However, the effect of a CPNB depends on the ability to place the catheter close to the nerve and that the catheter stays in place. Unfortunately, precise initial placement and secondary displacement are major challenges with existing catheter techniques [7–9] and may result in having no added benefit of CPNB compared to single-injection nerve block [10].

Femoral nerve block has been the preferred peripheral nerve block used for TKA. For continuous femoral nerve block both perpendicular and parallel (relative to the nerve) catheter insertion techniques are used. The two techniques seem to result in similar analgesia and opioid consumption after TKA, when successfully placed [11, 12]. However, inserting the catheter parallel to the nerve is more time consuming [12]. Furthermore, continuous femoral nerve block has also been shown to be an independent risk factor related to in-hospital falls after TKA [13] and the adductor canal block (ACB) has been proposed as an alternative with superior quadriceps strength and walking ability and the analgesic effects seems equal to that of a femoral nerve block [14].

**Fig. 1:**
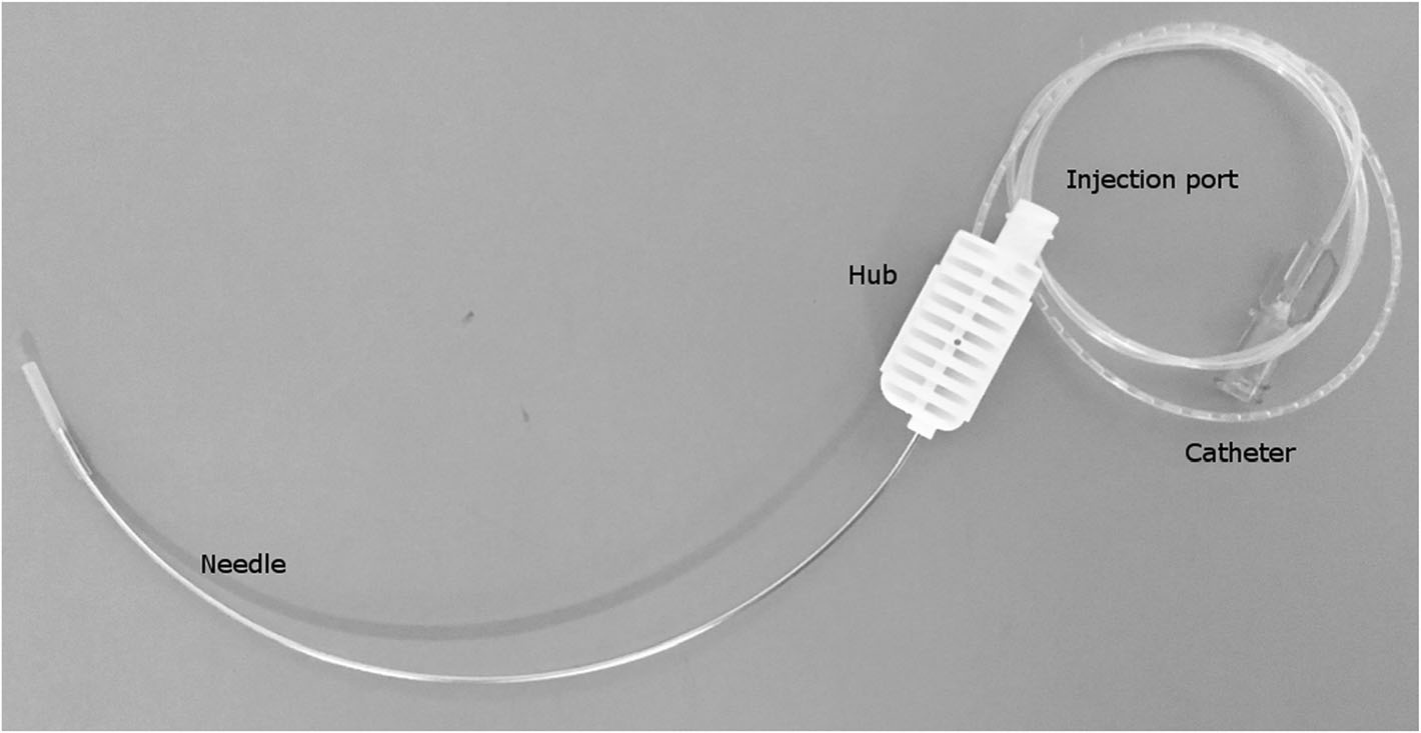
Illustration of the Suture-method catheter. The 19G nylon catheter is connected to the end of the needle. A detachable hub covers the connection. It has an injection port that allows injection through the needle. The proximal part of the catheter, closest to the hub, alternates in containing air and glue to increase echogenicity. The distal part of the catheter is patent with orifices at transition zone between proximal and distal part of the catheter. A Luer Lock injection port at the end of the catheter allows injection through the catheter

A new suture-like perineural catheter has been developed to overcome some of the challenges with existing catheters. The catheter (Fig. 1) has a curved needle with the catheter attached at the end of the needle. This enables precise ultrasound guided primary placement, with success rates close to 100% [15, 16]. Furthermore, with both ends of the catheter available for manipulation, it may be possible to reposition the catheter in case of displacement [17]. But before commencing with large scale randomized clinical trials, this remains to be investigated in in-vivo studies with different techniques to avoid potential confounding factors from the surgical procedure or pre-existing medical conditions.

Thus, in this pilot study we aimed to investigate the feasibility to reposition the suture-method catheter in the adductor canal (AC), for both perpendicular and parallel placement of the catheters.

## Methods

### Study design

The study was conducted as a randomized, blinded pilot study in 12 healthy volunteers from March to April 2017 at Nordsjællands Hospital, Denmark. The study was approved by the Regional Ethics Committee (H-16029530), the Danish Data Protection Agency and registered at clinicaltrials.gov (NCT03315481).

### Volunteers

Volunteers were recruited via a government-sanctioned website (sundhed.dk). All volunteers gave verbal and written informed consent before participation in the study.

The inclusion criteria were: age ≥ 18 years and American Society of Anesthesiologists (ASA) status classification 1 or 2. Exclusion criteria were: previous surgery, pain or neurological deficits in the investigated region; allergy to local anesthetics, pregnancy, breastfeeding or unwillingness to practice birth control during participation in the study.

### Randomization and allocation concealment

All volunteers had catheters inserted bilaterally. Primary randomization (1:1) determined catheter insertion using a perpendicular approach in one leg and parallel approach in the other leg. A secondary randomization dictated the direction of the intentional catheter displacement. Both randomization sequences were generated by a web-based randomization generator (sealedenvelope.com). The randomization lists and sequentially numbered opaque sealed envelopes were prepared by health personnel with no other involvement in the study.

### Blinding

All outcome assessments were performed by blinded investigators. However, the investigator who repositioned the catheter inevitably became unblinded to insertion technique during repositioning.

### Interventions

We performed a baseline sensory assessment of cold perception in the saphenous nerve cutaneous innervation area with an alcohol swab, obtained an intravenous access and monitored volunteers using continuous pulse oximetry, before inserting the suture-method catheters (Certa Catheter™; radius of curvature 75 mm, length 160 mm, Ferrosan Medical Devices, Szczecin, Poland). The skin and subcutaneous tissue at the insertion site was infiltrated with 3–5 mL local anesthetics (LA; lidocaine 1%). The catheters were placed bilaterally, by a single investigator, in the AC approximately midway between the anterior superior iliac spine (ASIS) and the patella [18, 19] using ultrasound (US) imaging (Edge system with HFL50 transducer, FujiFilm SonoSite, Netherlands). An in-plane, short-axis (SAX) US technique was used for catheter placement perpendicular in the AC advancing the needle from anterolateral to posteromedial. For catheter placement parallel in the AC, the needle was inserted in a proximal to distal direction, deep into the sartorius muscle using an out-of-plane SAX view and then an in-plane long-axis(LAX) view to enter the AC. Additional movie files show this in more detail [see Additional files 1 and 2]. The skin and subcutaneous tissue at the exit site was infiltrated with further 3–5 ml LA before penetration with the catheter needle. After placement, the catheters were fixated using Tegaderm™ dressings (3 M Healthcare, Copenhagen, Denmark). A bolus of 15 mL lidocaine 1% was administered through the catheters. The investigator assessed whether there was satisfactory spread of LA in the adductor canal as defined by Andersen et al. [20] Fifteen minutes after injection, a blinded research nurse performed the assessment of cold sensation using an alcohol swab on the medial part of the lower leg.

Once cold sensation returned, the investigator displaced the catheters according to the secondary randomization (toward entry or exit site of the catheter). After displacement, the same investigator measured the distance from the catheter orifice to the adductor canal using US. Measurements were repeated twice on each leg. If measurements were far apart, a third measurement was performed. Displacement distance was calculated as the average of these 2 measurements, a potential outlier measurement was excluded.

Subsequently, a second investigator assessed the catheter displacement distance. This assessment was blinded to the previous measurements performed by the first investigator. Once completed, the second investigator repositioned the catheters and LA was administered once more. The spread of LA was evaluated independently by the second investigator along with a third investigator during injection of LA. Assessment of cold sensation was repeated in the same manner as described above.

### Outcomes

Primary outcome was successful repositioning of the catheter, defined as a composite of US verified satisfactory spread of LA within the AC (assessment by second investigator) in combination with loss of cold sensation 15 min after the second injection of LA. Secondary outcomes were 1) Successful primary placement of the catheter, defined as a composite of US verified satisfactory spread of LA within the AC in combination with loss of cold sensation 15 min after the first injection of LA. 2) The estimation of limits of agreement between the investigators evaluating the distance from the catheter orifice to the AC after displacement. 3) The interrater agreement for satisfactory spread within the AC.

### Sample size estimation

Because of the exploratory design of the study, it was neither designed nor powered to show statistical differences between the two techniques. We assumed a successful catheter repositioning rate of 100% and wanted to establish a 95% confidence interval estimate of 75–100%. Thus, we calculated, that a sample size of 12 volunteers would be sufficient to assess whether the catheter could be successfully repositioned. We applied the Wilson interval method [21] for a more conservative 95% CI estimate.

### Statistical methods

Statistical analysis was performed using SPSS software (IBM SPSS Statistics, version 22.0.0; IBM Corp, Armonk, New York). Demographic data are presented as mean (SD). Primary and secondary outcomes are reported as proportions with 95% CI estimates, calculated using the Wilson interval method. A Bland-Altman plot was used to quantify the interrater agreement of displacement distances and Cohen’s Kappa statistics was applied to describe interrater agreement of LA spread.

## Results

**Fig. 2:**
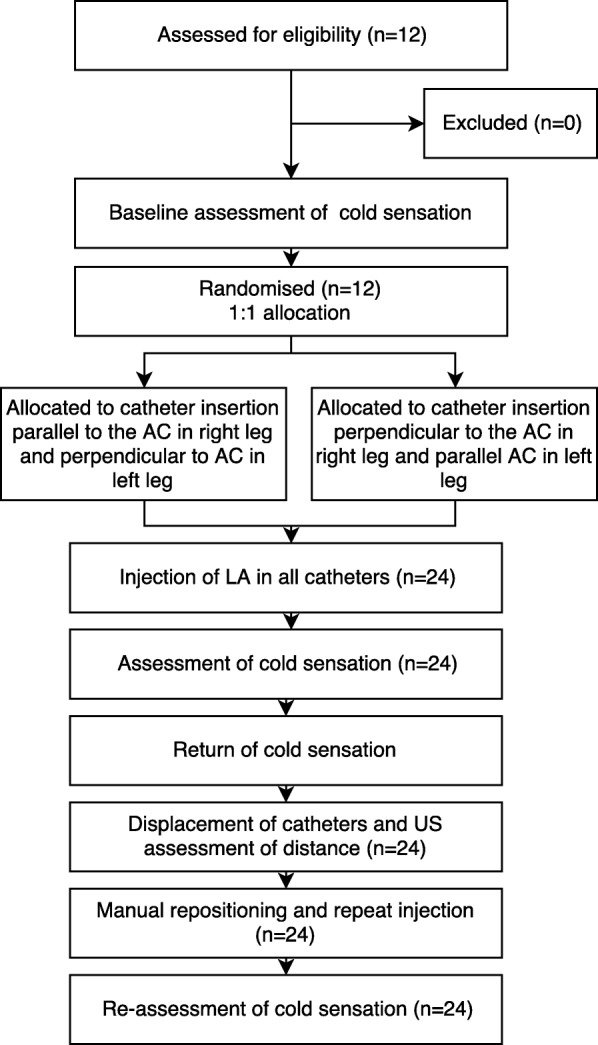
Modified CONSORT flow chart

Twelve volunteers were included in the study; all received the assigned interventions (Fig. 2). Demographic data for the volunteers are presented in Table 1.

**Table 1:** Demographics

	Mean	SD
Age (years)	22	3.5
Height (cm)	181	8
Weight (kg)	73	12
BMI (kg/m^2^)^a^	21	2.1

^a^BMI, body mass index

The rate of successful primary placement was 10/12 (83%; 95% CI [55–95%]) for catheters perpendicular to the AC, and 9/12 (75%; 95% [CI 47–91%]) for catheters parallel to the AC.

Of these, successful repositioning was achieved in 10/10 (100%; 95% CI [72–100%]) catheters placed perpendicular to the AC, and in 6/9 (67%; 95% CI [35–88%]) catheters placed parallel to the AC (Table 2). Individual level data for primary placement and repositioning are provided in the Additional file 3: Table S1.

**Table 2:** Primary placement and repositioning

	Perpendicular (*n* = 12)	Parallel (*n* = 12)
Successful primary placement proportions (95% CI)	10/12 83% (55–95%)	9/12 75% (47–91%)
*evaluated by spread*	11	10
*evaluated by loss of cold sensation*	11	9
Successful repositioning (primary outcome) proportions (95% CI)	10/10 100% (72–100%)	6/9 67% (35–88%)
*evaluated by spread*	10	8
*evaluated by loss of cold sensation*	10	6

95% CI: 95% confidence interval

**Fig. 3:**
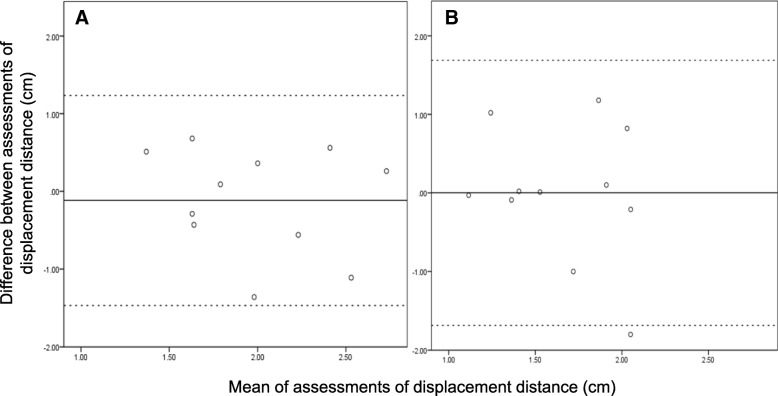
Bland-Altman plots for estimation of displacement distance for perpendicular and parallel insertion techniques. **a**: Perpendicular approach. **b**: Parallel approach

The estimation of limits of agreement between the investigators evaluating the distance from the catheter orifice to the AC after displacement is presented in Fig. 3 (Table 3).

**Table 3:** Secondary endpoint, estimation of limits of agreement between investigators assessing distance from orifice of catheter to AC

	Perpendicular (*n* = 11)	Parallel (*n* = 11)
Bias	−0.1173	0.0018
SD	0.6895	0.8607
Upper LoA	1.2342	1.6889
Lower LoA	−1.4688	−1.6853

*AC* adductor canal, *LoA* Limits of Agreement

Calculating interrater agreement for satisfactory spread within the AC, when attempting repositioning, proved impossible for catheters placed perpendicular to the AC, as one investigator rated all spreads satisfactory. However, investigators agreed on spread in 11 out of 12 cases (total agreement of 92%; 95% CI [65–95%]). For catheters placed parallel to the AC, the interrater agreement resulted in a Kappa value of 0.63.

Two volunteers had on their own opted to perform non-protocolled vigorous squatting with catheters in place. After study completion, both volunteers reported sensory deficits in the saphenous nerve cutaneous innervation area. They reported diminished sensation to touch and cold after participation. The sensory deficits subsided in one volunteer, the other volunteer reported minor deficits after 1 year. In the latter volunteer, the affected area initially involved the entire saphenous nerve cutaneous innervation area and had decreased by one third with a less clear delineation of the affected area. Furthermore, the volunteer had regained normal sensation to cold and pinprick but altered sensation to touch.

## Discussion

In this randomized, blinded pilot study, we were able to reposition a displaced suture-method catheter for both parallel and perpendicular insertions. The ability to place a catheter close to the targeted nerve is a major determinant in the subsequent success of the catheter. When using a traditional technique, the catheter is advanced through the needle and slightly beyond the needle tip. US visualization of the catheter tip is impeded by the catheter rarely remaining in the same plane as the US beam. Thus, hydrolocation or tissue movement is usually relied upon as a surrogate. However, the catheter orifice may often be located suboptimal with regards to infusions/repeated boluses regimens resulting in secondary failure. Furthermore, secondary failure of perineural catheters may be due to later displacement of the catheter, for instance due to increasing edema in the area or the patient being mobilized causing tissue movement. Several technical advances have been made to try and overcome these issues, but none permit repositioning, other than simple retraction [9]. The suture-method catheter is a new addition to the range of perineural catheters. The design of the suture-method catheter enables visualization of the catheter orifice. This provides opportunity to evaluate proper initial placement, later assessment of orifice position in case of displacement and the possibility of pulling at both ends, thereby enabling repositioning of the catheter. Presently, few data on its use has been published. We have previously shown that placing the suture-method catheter perpendicular to the nerve in the popliteal sciatic nerve block yields a high success rate and a low rate of displacement when using loss of cold sensation as indicator for success [16]. The repositioning qualities of the catheter has been examined in cadavers, and 42 of 43 catheters placed perpendicular to the nerves were successfully repositioned [15]. The present study provides the first in vivo evidence that a perineural catheter can be repositioned. The perpendicular placement seems to enable reliable repositioning in case of displacement as all catheters could be repositioned whereas catheters placed parallel to the AC could not be repositioned to the same extent. This may reflect that repositioning a catheter parallel to the ACB is technically more challenging. The anatomy of the AC involves several fasciae, which may be more difficult to distinguish with this technique. Further, visualization of the catheter in toto was more difficult with the parallel technique. Ultimately, repositioning was performed and final location was chosen based on visual assessment of LA spread. As our interrater agreement for satisfactory spread indicates, this is not perfect even though we used a well described definition of successful spread of LA in the adductor canal [20].

To further elucidate the technical challenges of repositioning for the two different techniques for catheter placement, we assessed how well agreement was between the individual catheters displacement distance. We believed a priori, that this marker served as a surrogate for how precisely the orifice could be identified, but it more likely reflects the variation from differences in US handling such as slightly different angling and tilting. We believe that the difference between limits of agreement for the perpendicular and parallel placement techniques reflect that it was more difficult to visualize the parallel catheter in its entire path within the AC creating increased variation.

We speculated that parallel placement would permit a longer catheter trajectory within the AC and would therefore be less prone to displace. However, we also speculated that the parallel approach would be technically more challenging with regard to primary placement and repositioning in case of displacement. To our knowledge, no data has been published on the superiority of the parallel versus the perpendicular placement of traditional ACB catheters. The two catheter techniques have been studied for other lower limb blocks with conflicting results [12, 22] and underline the complexity of the issue. Although it is tempting to transfer evidence from femoral nerve block catheter techniques, the potential difference in anatomy with regard to fascial layers, connective tissue and local response to surgical trauma make the AC unique.

Neither technique achieved our pre-specified lower limit for 95%CI estimate of 75% success rate for repositioning due to the small sample size, but taken together, the current study suggests that perpendicular placement enables more reliable repositioning in case of a displaced catheter.

Repositioning of a perineural catheter would result in improved pain relief for patients experiencing catheter failure, who could otherwise be subject to either opioid-based analgesia with adherent risks and side effects or repeated invasive regional analgesia with concomitant added risk of hematoma, infection or nerve damage. On the other hand, dual skin penetration and fixation of a suture-method catheter could, in theory, also expose the patient to the same risks. These risks can only be estimated from further studies and audits from clinical practice.

Of concern, two volunteers reported sensory deficits after study completion. Both subjects had on their own opted to perform non-protocolled vigorous squatting with catheters in place, reported pain during squatting but had chosen to carry on. We speculate that their sensory deficits were caused by the repeated pressure exerted on the saphenous nerve during forceful squatting.

There are several limitations to our study. We did not enroll patients undergoing surgery, which would have made our results more readily applicable to clinical practice. We chose healthy volunteers because it enabled us to eliminate confounding factors and several sources of random error that could potentially influence our results given our small sample size. Although patients undergoing surgery may differ from healthy volunteers in several aspects, we believe that our findings are applicable in a clinical context. Furthermore, we used distance as indicator of sufficient intentional displacement. During study planning, we chose not to administer LA through the displaced catheter to test for adequate displacement. This was because of concerns about local anesthetic systemic toxicity due to accumulated LA dosing. In theory, it is possible that the catheter would still be functional due to diffusion of LA along the catheter.

## Conclusions

This pilot study has provided valuable information on the feasibility to position and reposition a suture-method catheter using either perpendicular or a parallel placement technique. Our results suggest that the perpendicular placement provides higher chances of successful initial placement and repositioning for ACB. This enables informed choices for later randomized clinical trials.

## Additional files

Additional file 1: Catheter insertion parallel in the AC. US video of the parallel catheter placement technique. (MP4 20183 kb)

Additional file 2: Catheter insertion perpendicular in the AC. US video of the perpendicular catheter placement technique. (M4V 15669 kb)

Additional file 3: **Table S1.** Individual level data for primary placement and reposition. (DOCX 18 kb)

## Abbreviations

AC: Adductor canal
ACB: Adductor canal block
ASA: American Society of Anesthesiologists
ASIS: Anterior superior iliac spine
BMI: Body mass index
CI: Confidence interval
CPNB: Continuous peripheral nerve blocks
LA: Local anesthetic
LAX: Long-axis
LoA: Limits of Agreement
NRS: Numerical rating scale
SAX: Short-axis
SD: Standard deviation
TKA: Total knee arthroplasty
US: Ultrasound
