# Supplementary material for: Ultrasound guided repositioning of a new suture-method catheter for adductor canal block – a randomized pilot study in healthy volunteers
Source: BMC Anesthesiol. 2018 Oct 24;18:150. doi: 10.1186/s12871-018-0615-4 (PMC6201513; doi:10.1186/s12871-018-0615-4)
Supplement: Supplementary file 3 — Table S1. Individual level data for primary placement and reposition. (DOCX 18 kb) [file 12871_2018_615_MOESM3_ESM.docx]

## Table S1. Individual level data for primary placement and reposition.

| subject no. | Perpendicular | | | | | | Parallel | | | | | |
| --- | --- | --- | --- | --- | --- | --- | --- | --- | --- | --- | --- | --- |
|  | primary placement | | | repositioning | | | primary placement | | | repositioning | | |
|  | 1. satisfactory spread | 2. loss of cold sensation | **agreement between 1+2** | 1. satisfactory spread | 2. loss of cold sensation | **agreement between 1+2** | 1. satisfactory spread | 2. loss of cold sensation | **agreement between 1 + 2** | 1. satisfactory spread | 2. loss of cold sensation | **agreement between 1 + 2** |
| 1 | + | + | **+** | + | + | **+** | + | - a) | **-** | + | + | **+** |
| 2 | + | + | **+** | + | + | **+** | + | + | **+** | + | + | **+** |
| 3 | + | + | **+** | + | + | **+** | - | - | **+ *)** | + | - | **-** |
| 4 | + | + | **+** | + | + | **+** | + | + | **+** | + | + | **+** |
| 5 | - | + | **-** | + | + | **+** | + | + | **+** | - | - b) | **+ *** |
| 6 | + | - c) | **-** | - | - a) | **+*** | + | + | **+** | + | - a) | **-** |
| 7 | + | + | **+** | + | + | **+** | + | + | **+** | + | + | **+** |
| 8 | + | + | **+** | + | + | **+** | + | + | **+** | + | - b) | **-** |
| 9 | + | + | **+** | + | + | **+** | + | + | **+** | + | + | **+** |
| 10 | + | + | **+** | + | + | **+** | + | + | **+** | + | + | **+** |
| 11 | + | + | **+** | + | + | **+** | - | - | **+ *)** | + | + | **+** |
| 12 | + | + | **+** | + | + | **+** | + | + | **+** | + | + | **+** |
| a) loss of cold sensation after 30 minutes b) altered sensibility, but not loss of cold, after 15 minutes (not examined at 30 minutes)  c) altered sensibility, but not loss of cold, after 30 minutes  *) no satisfactory spread, no loss of cold - agreement on dysfunctional catheter | | | | | | | | | | | | |
